# Supplementary material for: Lipomatous Tumors: A Comparison of MRI-Reported Diagnosis with Histological Diagnosis
Source: Diagnostics (Basel). 2022 May 21;12(5):1281. doi: 10.3390/diagnostics12051281 (PMC9141562; doi:10.3390/diagnostics12051281)
Supplement: Supplementary file 1 [file diagnostics-12-01281-s001.zip › diagnostics-1660228-supplementary.pdf]

histologic entity	frequent false diagnosis in MRI	n	total in cohort	false in %	total false per entity in %
lipoma	soft tissue sarcoma	3	116	2.6	
lipoma	LPS	21	116	18.1	
lipoma	malignancy cannot be excluded	4	116	3.5	
lipoma	ALT	11	116	9.5	
lipoma	lymphoma	1	116	0.9	34.5
fibro lipoma	LPS	1	4	25.0	
fibro lipoma	malignancy cannot be excluded	1	4	25.0	50,00
spindle cell lipoma	LPS	1	3	33.3	
spindle cell lipoma	malignancy cannot be excluded	1	3	33.3	66.7
angio lipoma	LPS	1	6	16.7	16.7
hibernoma	malignancy cannot be excluded	2	3	66.7	66.7
ALT	LPS	4	39	10.3	
ALT	rhabdomyosarcoma	1	39	2.6	12.8

Supplementary Data: Entities of over-diagnosed tumours in MRI. Altogether, 52 lipomatous tumours were wrongly categorized as more malignant in MRI than in histologic assessment.
